# Interface Pressure System to Compare the Functional Performance of Prosthetic Sockets during the Gait in People with Trans-Tibial Amputation

**DOI:** 10.3390/s20247043

**Published:** 2020-12-09

**Authors:** Salvador Ibarra Aguila, Gisel J. Sánchez, Eric E. Sauvain, B. Alemon, Rita Q. Fuentes-Aguilar, Joel C. Huegel

**Affiliations:** 1 Biomechatronics Laboratory, School of Engineering and Science, Tecnologico de Monterrey, Zapopan, Jalisco 45201, Mexico; A01630811@itesm.mx (S.I.A.); giseljss95@gmail.com (G.J.S.); A00829695@itesm.mx (E.E.S.); beatriz.alemon@tec.mx (B.A.); or jhuegel@mit.edu (J.C.H.); 2 Center for Extreme Bionics, Massachusetts Institute of Technology, Cambridge, MA 02139, USA

**Keywords:** pressure interface, prosthesis evaluation, measuring interface, pressure distribution, socket interface, socket comfort, peak pressures, transtibial, amputation, socket quantitative evaluation

## Abstract

The interface pressure between the residual limb and prosthetic socket has a significant effect on the amputee’s mobility and level of comfort with their prosthesis. This paper presents a socket interface pressure (SIFP) system to compare the interface pressure differences during gait between two different types of prosthetic sockets for a transtibial amputee. The system evaluates the interface pressure in six critical regions of interest (CROI) of the lower limb amputee and identifies the peak pressures during certain moments of the gait cycle. The six sensors were attached to the residual limb in the CROIs before the participant with transtibial amputation donned a prosthetic socket. The interface pressure was monitored and recorded while the participant walked on a treadmill for 10 min at 1.4 m/s. The results show peak pressure differences of almost 0.22 kgf/cm${}^{2}$ between the sockets. It was observed that the peak pressure occurred at 50% of the stance phase of the gait cycle. This SIFP system may be used by prosthetists, physical therapists, amputation care centers, and researchers, as well as government and private regulators requiring comparison and evaluation of prosthetic components, components under development, and testing.

## 1. Introduction

**Figure 1:**
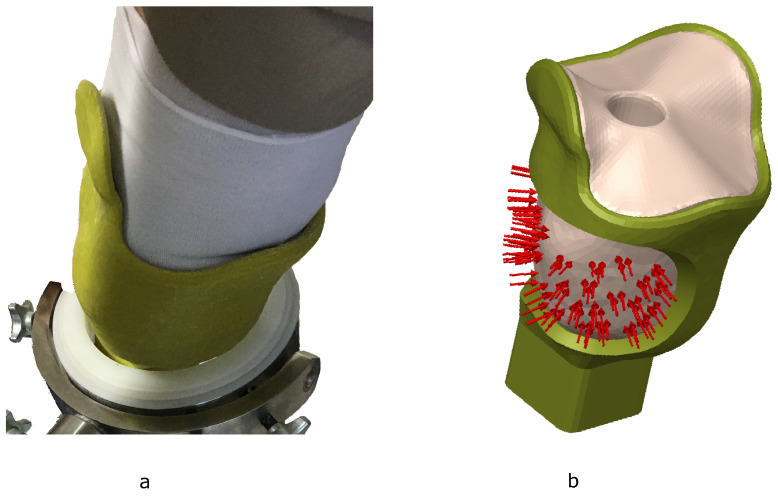
Interface pressure (IP) between lower limb residuum and socket. (**a**) 3D printed socket being tested by an amputee in an aluminum support block. (**b**) Cutout 3D model showing the pressure exerted by the prosthetic socket over the interface area of the residual limb.

The pressure exerted by a prosthetic socket over the residual limb is known as interface pressure (IP); see Figure 1. The IP has a significant effect on the amputee´s mobility, comfort and level of satisfaction about their prosthesis. A critical factor in the success of the functional performance of a prosthesis is the physical connection to the human body formed by the bio-mechanical interface system. Bio-mechanical interfaces are mechanical structures that form the connection between a device and a tissue region, thereby aiming to minimize tissue discomfort and injury, while still adequately transferring loads and forces back and forth from the musculoskeletal system to the prosthesis. For transtibial amputees, this bio-mechanical interface typically consists of a prosthetic liner and socket which together aim to provide a quality fit. A quality fit can be defined as the ability to provide comfort and appropriate load distribution [1].

Lower limb prosthetic users exert and feel pressure between the socket and lower limb during daily activities. The underlying soft tissues and skin of the residuum are not accustomed to weight bearing; thus, there is the risk of various skin problems, such as tissue ulcers, follicular hyperkeratosis, allergic contact dermatitis, infection, and veracious hyperplasia because of constant or repetitive peak pressures applied by the transtibial socket [2,3]. It is well known that the socket design plays a significant role in determining the quality of the fit between the prosthesis and the residual limb [2]. Therefore, some researchers have prioritized an improvement to the quality of fit via socket re-design and seek to employ interface pressure (IP) as a direct and quantitative means of evaluation. These studies sense the IP using force sensing resistors (FSR) or pressure transducers [4,5]. Currently, there are several systems for measuring IP between a subject and a support surface, such as the residual limb and prosthesis. These systems have different types of sensors, namely capacitive, piezo resistive, and pneumatic [6,7,8,9]. The Tekscan brand offers products and sensors for pressure measurement. One of the Tekscan products is the F-Socket, which is capable of measuring IP to compare, in a clinical investigation, the performance of several trans-tibial sockets and liners during the gait cycle [10,11,12]. As we mentioned before, the quality of fit between the socket and the residual limb depends on the transferring loads from the musculoskeletal system to the prosthesis. Those loads in specific areas of anatomical zones, including the tibia and fibula head regions, could be monitored, thereby sensing contact pressures in stance phase and during gait. Analyzing the peak pressures and the differences between pressures in different regions of interest could be employed to evaluate and compare the quality of fit and the functional performance of prosthetic sockets. Sengeh et al. showed that the contact pressures over the fibula and tibia anatomical landmarks were different during the gait in a comparison study of the design of two different sockets [12].

### The Gait Cycle on the Amputee and the Residuum

**Figure 2:**
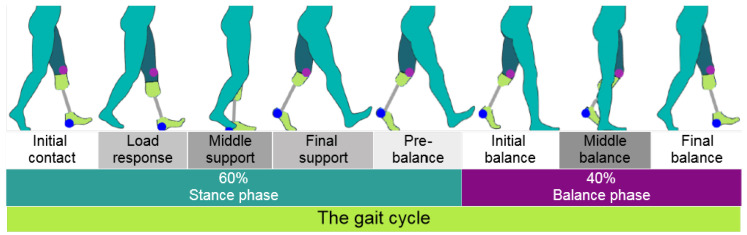
The two main gait cycle phases with the key support and balance subphases of the residual left lower limb with a transtibial prosthesis.

According to the characteristics and key events of a typical gait cycle reported by Mendoza et al., the gait cycle can be normalized by time or by length, thereby establishing percentages for each phase between key events. From the 100% of a gait cycle, 60% corresponds to the stance phase, while the remaining 40% corresponds to the swing phase [13]. The stance phase begins at the starting point of the gait, easily identified by the heel strike on the floor or initial contact as shown in Figure 2 and ends in the pre-balance or pre-swing phase when the metatarsus is about to lift off the ground. On the other side, as seen in Figure 2, the balance or swing phase starts when the toe is off the floor and ends when the heel is about to strike the floor, starting the cycle all over again. When a foot is in the stance phase, the other is in the swing phase with a very small overlap [14].

For transtibial amputees, the stance phase over the prosthetic foot is the one of interest because, during this phase in the initial contact, the prosthetic foot makes total contact with the floor, starting the load transfer from the other foot, continuing with the middle support, which applies the full load onto the prosthetic foot as the non-amputated limb lifts off the floor. At this time, almost complete body weight is applied, resulting in a pressure distribution across the entire residuum-socket interface. This continues until the load is removed during the final balance [13]. It is well known that the body weight creates pressure between the residuum and the socket of the prosthesis, a pressure that results in pathological gaits of the amputee due to many causes but primarily due to excessive knee flexion during the stance phase or insufficient knee flexion due to discomfort in the distal anterior region of the residuum. To avoid the pain, the amputee shortens the step length, increases back bending, mimicking a hunched back, resting the heel on the ground with increased activity of the hip extensor or all of the above [15].

**Figure 3:**
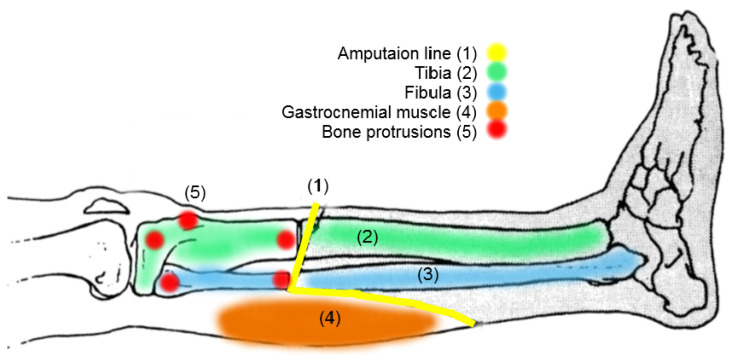
Amputation line for the transtibial amputee showing residual anatomy points of pressure over soft tissue. The bone protrusions include tibial and fibula epicondyle, head of the tibia, and distal amputation line for the tibia and fibula noted in red points (based on Reference [15]).

This discomfort may not end only in a distorted or pathological gait, but in damage to the soft tissue due to the anatomy of the transtibial amputee residuum, as shown in Figure 3. The amputation of the tibia and fibula creates two new bone bulges on the residuum below the soft tissue of the gastrocnemius muscle. This muscle is not activated any longer; rather, it is used to create the pad below the tibia and the fibula and closing the residual limb. The residuum resulting from the transtibial amputation is cylindrical and well padded, thanks to the skin lap and gastrocnemius muscle, to avoid bone protrusion discomfort; however, it can still happen because of the residual tibia and fibula of the residuum, where bone bulges are clearer [15,16].

As Figure 3 shows, the most relevant bone bulges that are subject to high pressure inside the prosthetic socket, are the medial and lateral condyles of femur, the head of the fibula [17], and the distal cuts from the tibia and fibula which are around critical points of pressure reference called critical region of interest (*CROI*).

The aim of this study was threefold:

- To compare the performance of our socket interface pressure (SIFP) device versus the F-Socket due that both of the them are products able to evaluate the functional performance of prosthesis sockets.
- To evaluate the quality of fit and the functional performance of prosthesis sockets during the gait using the stump/socket pressure distribution of a unilateral amputee by means of a integrated socket interface pressure system. The system comprises a Kinect motion capture system, a portable SIFP device (Bluetooth-enabled), and a MATLAB^®^ code. The system senses the pressure at the CROI of the residual lower limb during the gait and displays pressure graphs and gait graphs in real time.
- To detect via the sensitive range of the 6 sensor array, variations between sockets and pressure values at the CROI which could lead to detect pain points that negatively affect posture and gait of the amputee.

This paper is organized as follows: Section 2 presents the materials and methods, including equipment, motion capture sub-system, comparison with the F-Socket, and the experimental test procedure; Section 3 presents the results, including those for the sensor characterization, comparison test, and SIFP system test, followed by the Discussion and Conclusion sections.

## 2. Materials and Methods

**Figure 4:**
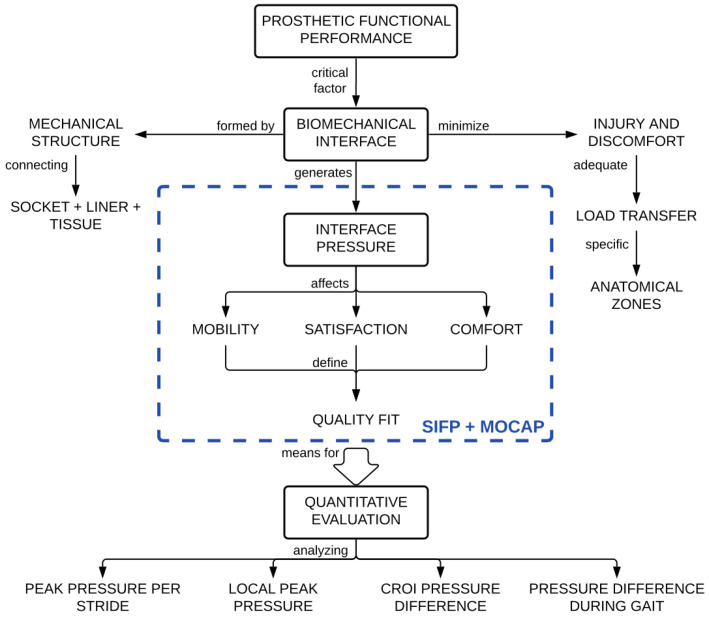
Conceptual Diagram of the problem and proposed socket interface pressure (SIFP) system shown in blue dashed line. It includes both the SIFP device and the motion capture (MOCAP) sub-system.

One of the critical factors in determining the functional performance of a prosthesis is the bio-mechanical interface that occurs between the amputee’s residuum and the mechanical structure of the manufactured socket. This interface necessarily generates a pressure distribution defined from both the mass/weight and inertia of the user and the total surface area of the socket. This pressure distribution directly affects mobility, satisfaction, and comfort. The SIFP system, as shown in Figure 4, is comprised of two subsystems: the SIFP device and the motion capture sub-system, both shown within the blue dashed box. Together, they serve as a means to quantitatively evaluate the quality of fit by identifying peak pressures and pressure differences. The materials and methods employed are presented as follows: equipment and sensor characterization, subject selection, and bio-mechanical marker location, followed by presentation of the motion capture subsystem. Then, the methods for the two tests are further detailed, namely the comparison between the F-Socket and SIFP device and the subject test procedure.

### 2.1. Materials and Equipment

**Figure 5:**
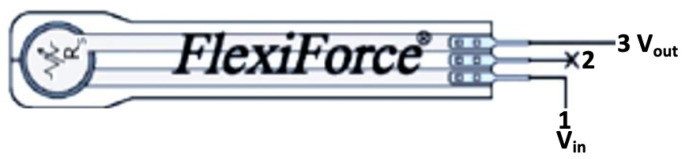
Resistive sensor FlexiForce A201^®^ connection diagram, where node 1 refers to input voltage, and node 2 refers to ground and node 3 refers to output voltage.

**Figure 6:**
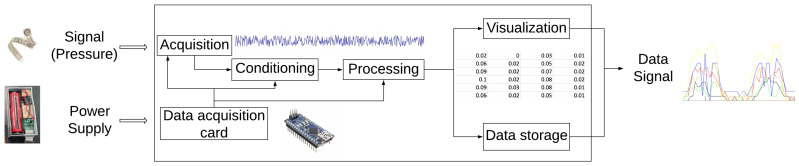
Schematic diagram of the socket interface pressure (SIFP) device.

The sensor employed for the system can be seen in Figure 5. The functional diagram of the system developed to build the SIFP device is shown in Figure 6.

The power supply of the device is a 3 V battery of 2500 mAh. This supply includes a switch to turn on/off the system. The input of the system is the signal transmitted by the resistive type pressure sensor that sends values between 0–5 V (see Figure 6).

A resistive type pressure sensor (Flexi Force A201^®^) was selected to use in the SIFP system because of its flexibility and detection area, its simplicity of connection and its linearity in its range between 95% and 98%. The pin connection of the sensor is shown in Figure 5.

Data received are processed to get the corresponding pressure generated in the contact area then those values are plotted in MATLAB^®^’s graphing interface, where the pressure generated can be seen as the output.

For the pressure data acquisition, it was decided to use an Arduino^®^ micro controller, which has the following specifications that were taken into account for the device: 12 digital I/O pins, 5–12 V input voltage, 5 V output voltage, Maximum total output current of 150 mA, Micro-USB connector for programming, 4-pin per 10-bit ADC, Rx and Tx Hardware Serial Connections, and an HC-05 Bluetooth Module. On the part of the conditioning and processing, a signal amplification stage is carried out which follows the configuration of an inverted amplifier. Following the data sheet of the sensors, the recommended design by the manufacturer is followed, where the sensors are supplied with the negative voltage as indicated in the data sheet.

From the circuit recommended by the manufacturer, it is important to consider the reference resistance (Rf), which controls the sensitivity/range trade-off of the sensor, in which resistance varies in a range of 1 KOhm to 100 KOhms, determining, if the value is low, decreases in the sensitivity of the sensors, while increasing the range or in the case of increasing resistance also increases sensitivity but decreases the range. For this solution, a resistance that varies up to 10 KOhms was proposed in order to provide a sensitivity of 5 kgf/cm${}^{2}$ Likewise, for the operational amplifiers, the OpAmps (MCP 6004) recommended by the manufacturer were used.

The data acquisition card was used to perform the analog-digital conversion and communication with the computer which handles the interface. On the part of the graphical interface, a code was programmed in MATLAB^®^ which can receive and graph the data of the signals sent by the Arduino’s Bluetooth module.

In this way, with the MATLAB^®^ user interface, the pressures generated in each gait cycle can be visualized almost in real-time. These pressures are generated in units of kgf/cm${}^{2}$ that can also be converted easily to kPa as needed, given that 1 kgf/cm${}^{2}$ = 98.0665 kPa. These units were selected in order to get a better comprehension of the interaction of the force exerted by the body weight over the socket surface, as kilogram-force over area provides a more intuitive understanding of the system to measure.

#### Pressure System Characterization and Linearization

**Figure 7:**
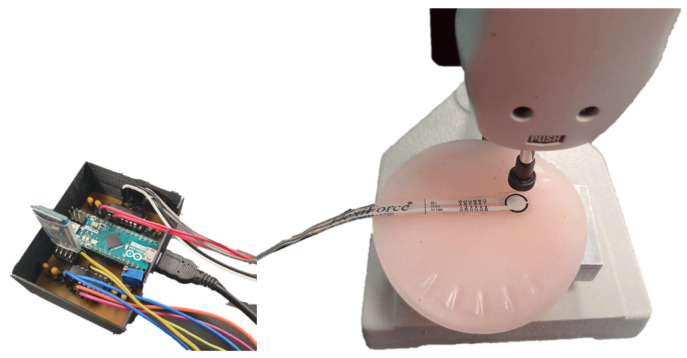
Characterization of SIFP via dynamometer applying various loads to a soft elastomeric material that has similar physical properties as a soft human tissue.

For the sensor system characterization, a dynamometer was used to apply a known force on the respective sensor’s area, an circular area of 10 mm in diameter. The forces were applied on a round piece of elastomeric material to simulate the reaction forces of the soft tissue of human skin to the compressed forces from the socket, as shown in Figure 7. The use of a soft material for the characterization of the SIFP system is important because it has similar elastic properties to those of human skin and experiences a similar deformation when a force is applied to it.

For every sensor, a mass between 0 to 2 kg was applied covering the totality of the sensor´s area. The steps used for static characterization and linearization of the system were the following:

1. Place 1/3 of the test weight on the sensor. Leave the weight on the sensor for approximately 3 s then remove the weight from the sensor.
2. Place 2/3 of the test weight on the sensor. Leave the weight on the sensor for the same amount of time then remove the weight from the sensor.
3. Place the full test weight on the sensor. Wait the approximate amount of time again then remove the weight from the sensor.

**Figure 8:**
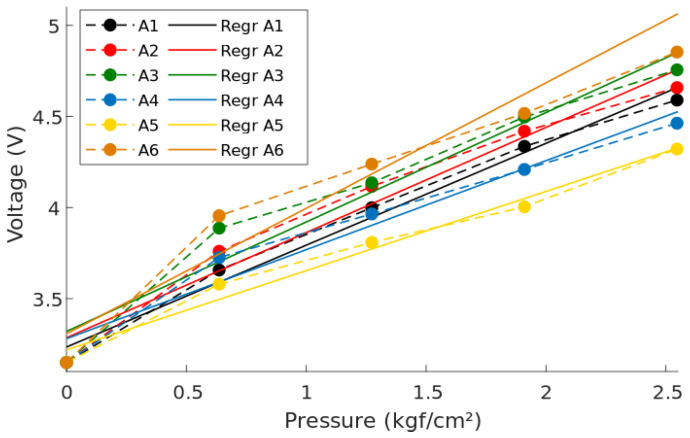
Linearization of the six pressure sensors. The dotted lines represent the obtained voltages, and the solid lines represent each sensor’s linear regression.

Using the known forces of each trial and the area of the sensors, the pressure was calculated in kgf/cm${}^{2}$ using the formula $P=\frac{F}{A}$ with A=0.7854 cm${}^{2}$. The voltage output at each applied force was recorded to linearize the system, using a linear function. Figure 8 shows the test voltage output and regression line for each sensor distinguished by color.

The linear functions are implemented into the microcontroller for data acquisition, to transform the voltage output of the device into pressure data. A reference voltage was established for the analog to digital conversion with a value of 645 bits. The reference in Volts (*y*) can be calculated using $y=\frac{5x}{1024}$, where *x* is the input signal in bits. The device does not need further calibration once the system has been linearized. It uses the characterization data as a reference in order to avoid the need to calibrate the device before each test.

### 2.2. Subject Selection and Protocol

Although the overall study will recruit 16 participants and has been approved by an ethics committee (Hospital la Misión S.A de C.V. (PR2018-17 Huegel)), the current pilot trial study only has one participant. The participant of this research study is an unilateral transtibial amputee. He is 43 years old, with more than one year since the amputation procedure and without any other musculoskeletal condition, nor any other cardiovascular, pulmonary, or neurological disorder. He has physical activity of at least K3 (candidates with the ability or potential to walk with variable cadence, capable of performing exercises with the prosthesis beyond simple locomotion [18]). Exclusion criteria for the overall study include:

- Candidates whose residual limb shows significant morphological changes during the study.
- Candidates that have the presence of metal implants or unsafe conditions for imaging studies or that prevent a CT study.

### 2.3. Croi Location

**Figure 9:**
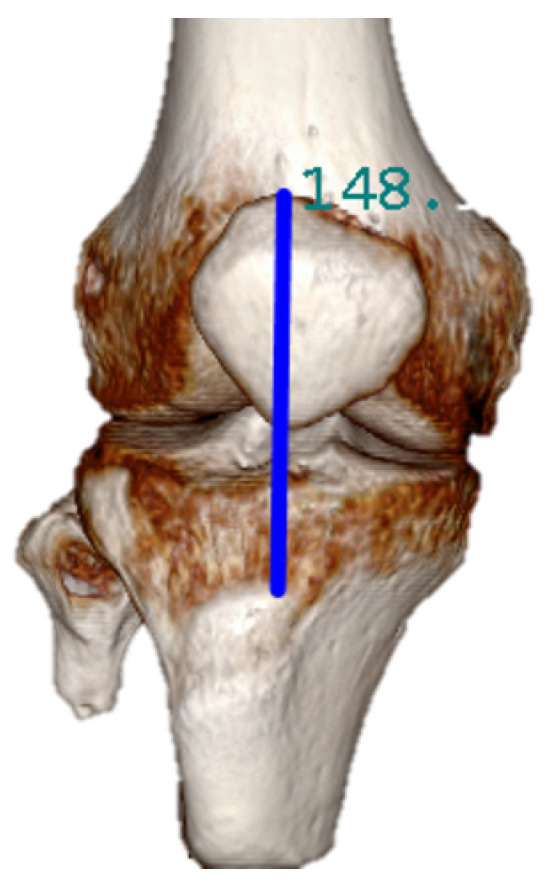
Three-dimensional CT image of the transtibial amputee participant.The measurement to localate the first critical regions of interest (CROI) is taken from the superior point of the patella to the middle point of the tibial crest.

The position of the CROI is defined by directly measuring from the CT image obtained from the residual lower limb of the participant, as shown in Figure 9.

The distance between the knee and the tibial crest is particularly different for each person. CT images allows to take more precise measurements than those taken via the traditional palpation technique. This distance represents the position of the first CROI. The following CROI are placed radially at 60°, 120°, 180°, 240°, and 300°. The overall research study approved by the ethics committee also uses the CT images to improve the socket design for transtibial amputee; therefore, in the current trial study, we could to use them for the location of the CROI.

### 2.4. Motion Capture Subsystem

#### 2.4.1. Parameters to Measure

The motion capture subsystem is a means to know the gait cycle, as well as the phases where the participant is at each moment wearing the SIFP device during a ten-minute walk on a treadmill at 1.4 m/s.

The parameters to measure with the motion capture subsystem are necessarily **both the ankle and knee position on a lateral plane** **(*x,y*); the frame is rotated 90° so that the anatomical saggital plane corresponds to the plane** **(*x,y*)** of the camera, since those joint positions are crucial to know the gait cycle phase the participant is in while walking.

#### 2.4.2. Materials and Setup for Motion Capture

For the motion capture subsystem, the Kinect sensors V1 and V2 were selected for their low cost as compared with other motion capture systems, such as *Nexus* software, with *VICON* cameras as depth sensors. Kinect was also selected for the extensive documentation in *Mathworks* for Kinect V1 and V2 as motion capture devices; therefore, MATLAB^®^ was selected as the software for saving and processing the data obtained from the Kinect sensors. One of each type was employed due to hardware availability. The complete motion capture system is listed below:

- Kinect v2 -Body Tracking Setting
- Kinect v1 for Windows -Skeleton Tracking Setting
- Treadmill NordicTrack^®^ C97OPRO
  - Configurable and maximum speed of 19.2 km/h
  - Units of measure of the International System
  - 60”X 20” track
  - Front and side support handles
  - Screen that includes speed display, distance traveled, and travel time
  - Control keypad
- 1 Workstation Precision 5820 Tower
  - Intel Xeon Processor W family
  - Graphic card Radeon Pro WX
  - 16GB RAM
  - Operating System Windows 10.
- Software
  - Kinect^®^ for Windows Developer Toolkit V1.8
  - Kinect SDK
  - Microsoft Speech Platform SDK
  - MATLAB^®^ R2018b
    * Image Acquisition Toolbox Support
      Package for Kinect for Windows Sensor
    * Microsoft Kinect for Windows Support
      from Image Acquisition Toolbox

**Figure 10:**
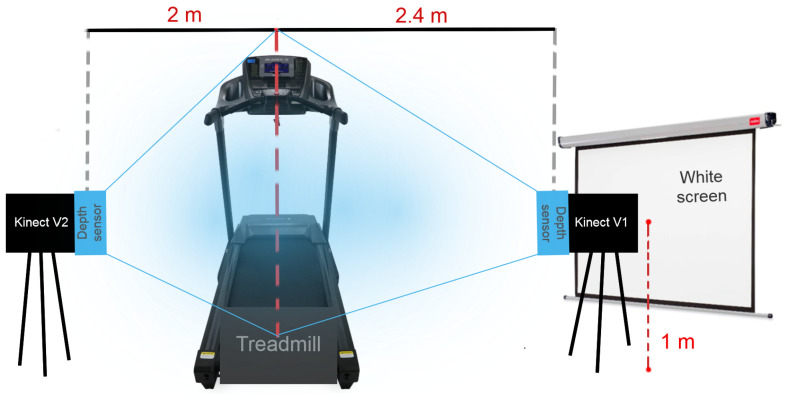
Motion capture system space setup design.

In order to keep the Kinect V1 and V2 measurements as precise as possible, the distance from the target (the centerline of the treadmill) to the Kinect sensors of each of the two versions is set to two meters according to the Kinect hardware specifications. The vertical field of view for the Kinect depth sensor V1 is of 40° and for the V2 it is of 60°. To be able to fit an average height individual on both sensor´s vertical field of view, the Kinect sensor V1 was placed 40 cm further away over the already established two meter distance from the centerline of the treadmill, as seen in Figure 10.

#### 2.4.3. Software Algorithm in MATLAB^®^ for Motion Capture

**Figure 11:**
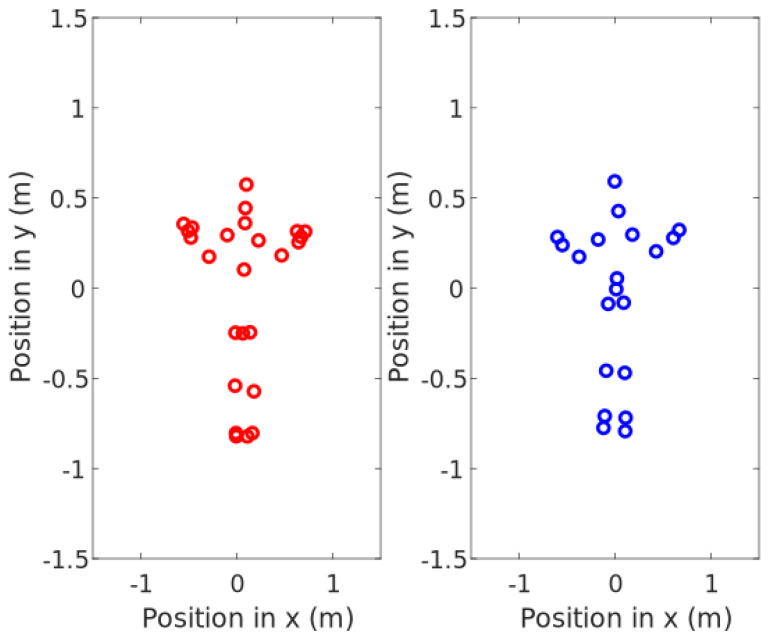
Joint coordinate projection for subject detection before 90${}^{\circ }$ turn for lateral motion capture of Kinect V2 and V1 on (*x,y*) plane.

To visualize ankle and knee position, let the knee and foot coordinates be saved in a three-dimensional array of (*x,y,z*) coordinates along with the rest of the 20 and 25 key joints Kinect V1 and V2 return per frame. A visualization of the key joints is shown in Figure 11 that plots them in real time as the program starts.

### 2.5. Comparison between F-Socket and SIFP

**Figure 12:**
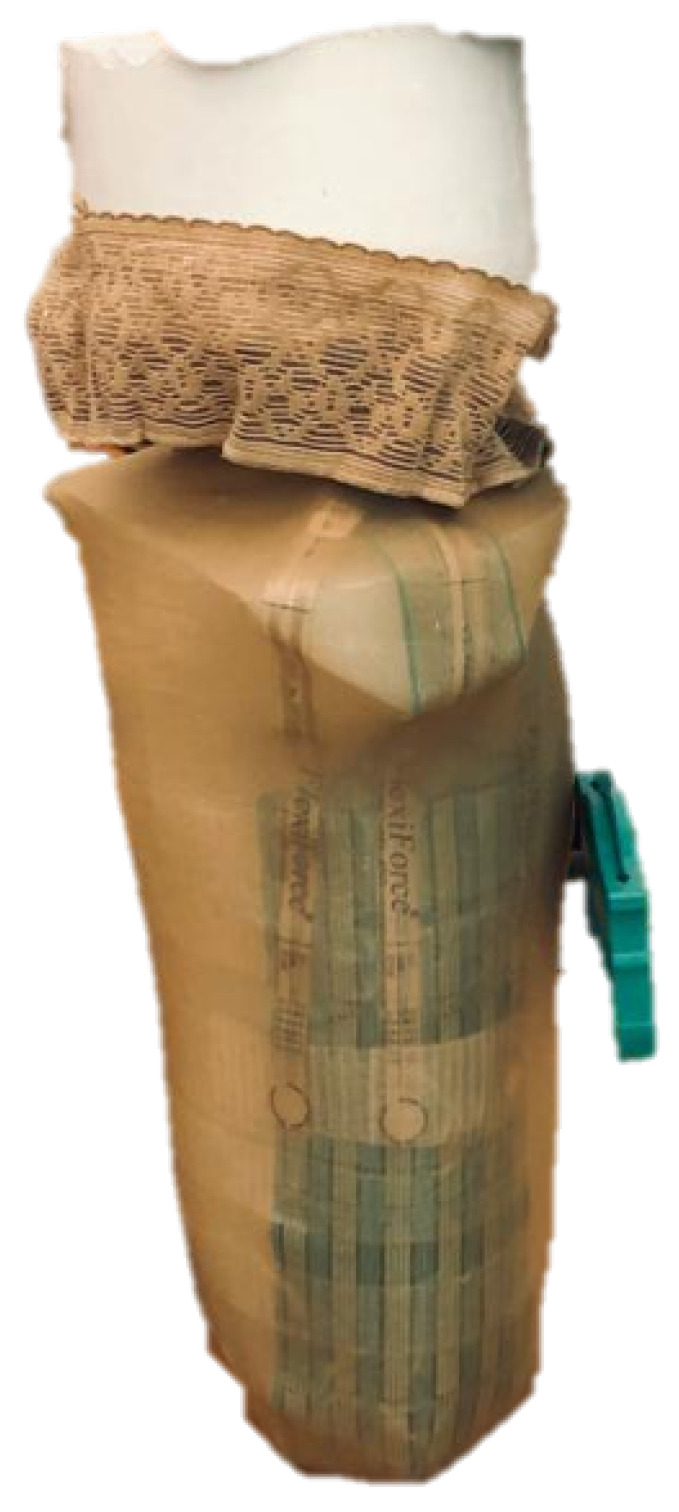
Artificial residuum with the socket interface pressure (SIFP) sensor and F-Socket sensors before the socket is installed.

The objective of comparing the F-Socket system to the socket interface pressure (SIFP) device developed in this research is to validate the accuracy of loads detection by both systems. F-Socket System consists of an array of pressure sensors forming a matrix, capable of mapping pressure distribution around an object. A test was conducted involving both systems to compare the results. Figure 12 illustrates this test where the sensors of SIFP device were placed on top of the F-Socket according to CROI locations, and this in turn was placed in a synthetic residuum made of elastomeric material.

**Figure 13:**
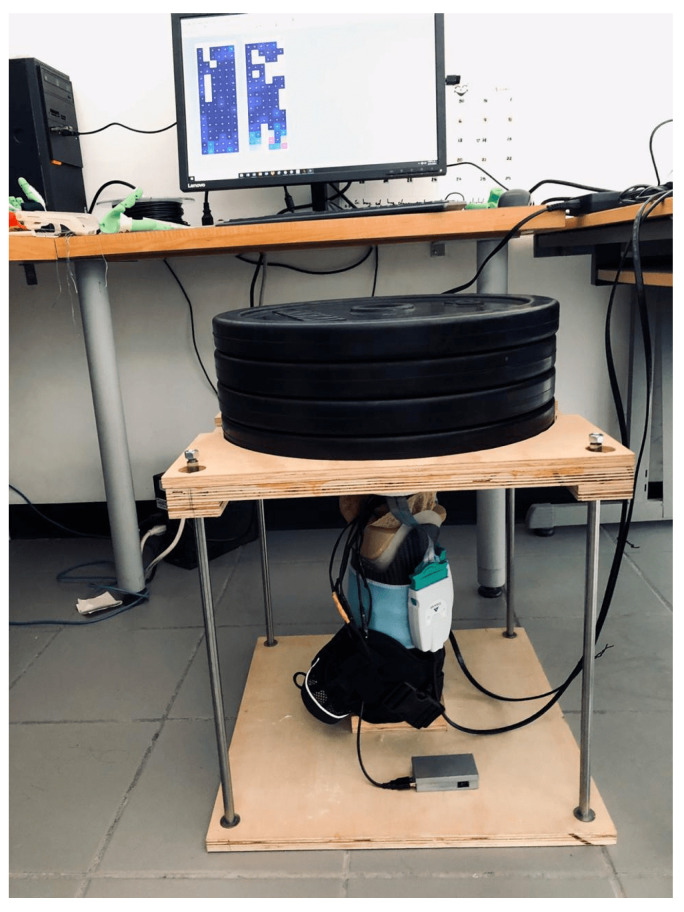
Platform for the comparative test between SIFP device and F-Socket, applying up to 80-kg loads, in 20-kg increments, to the artificial residuum. The SIFP device and F-Socket sensors were installed between the artificial residuum and the socket.

Loads were applied to the artificial residuum in order to simulate the walking protocol followed during subject testing. Loads from 0 to 80 kg (see Figure 13) in 20 kg increments was followed by unloading back to zero also in −20 kg increments.

Ten iterations were performed to have sufficient data for the comparison of both tests. Five iterations applying the loads simulating an loading cycle (such as heel strike) and another five iterations removing the loads simulating an unloading cycle (such as toe off).

The comparison between the load levels around the residuum registered by the two systems indicates that the uneven distribution of loads within the socket is detected accurately by both systems.

### 2.6. Subject Test Procedure

Before the test began, the participant read the protocol and signed the consent form. The test procedure consists of the following steps:

1. The prosthetic socket is removed by the participant or with help.
2. The SIFP device is placed on the participant and fitted to his waist.
3. The CROIs are identified with non-permanent marks and the sensors of the SIFP device are placed at the CROIs, as shown in Figure 18.
4. A nylon stocking is placed on the residual limb and over the sensor arrangement in order to prevent possible sensor motion inside the socket.
5. The prosthetic socket to be tested is donned onto the residual limb by the participant or with help.
6. A pressure sensing test is performed with the participant at rest sitting in a chair.
7. The participant proceeds to perform a physical activity (walk on a treadmill) for 10 min at 1.4 m/s as follows:
  (a) The motion capture program is started.
  (b) The participant is asked to step onto a yellow mark on the floor facing the Kinect and wave with both hands in order to get detected by the camera. If the participant is not detected by the camera, the data will not be saved, and the legend ***SUBJECT NOT DETECTED*** will pop up.
  (c) The participant is now asked to stand on the treadmill facing to the Kinect and wave again towards it. By now, the camera should have detected the participant. If so, in the central figure, the points of the coordinates of the camera will appear in a central figure projecting the participant’s movement in real time, as seen in Figure 11.
  (d) The stopwatch is started as soon as the treadmill is set on the 1.4 m/s speed, and the participant is walking comfortably.
  (e) The participant walks for ten minutes, trying to remain as straight as possible and trying to replicate his normal gait.
  (f) When the stopwatch hits the ten-minute mark, the data capture is stopped to be recorded, and the work space of MATLAB^®^ is saved.
8. The participant gets off the treadmill, removes the prosthetic socket, and removes the pressure sensors.
9. All the equipment is removed from the residuum of the participant.

In order to compare the functional performance of another type of socket, the participant takes a 10-min break and rest. After that period of time the sensors of SIFP device are placed in the CROI on the residuum, stocking in place, and the prosthetic socket to be tested is donned for the test, and the test procedure begins again.

## 3. Results

### 3.1. Static Characterization

**Figure 14:**
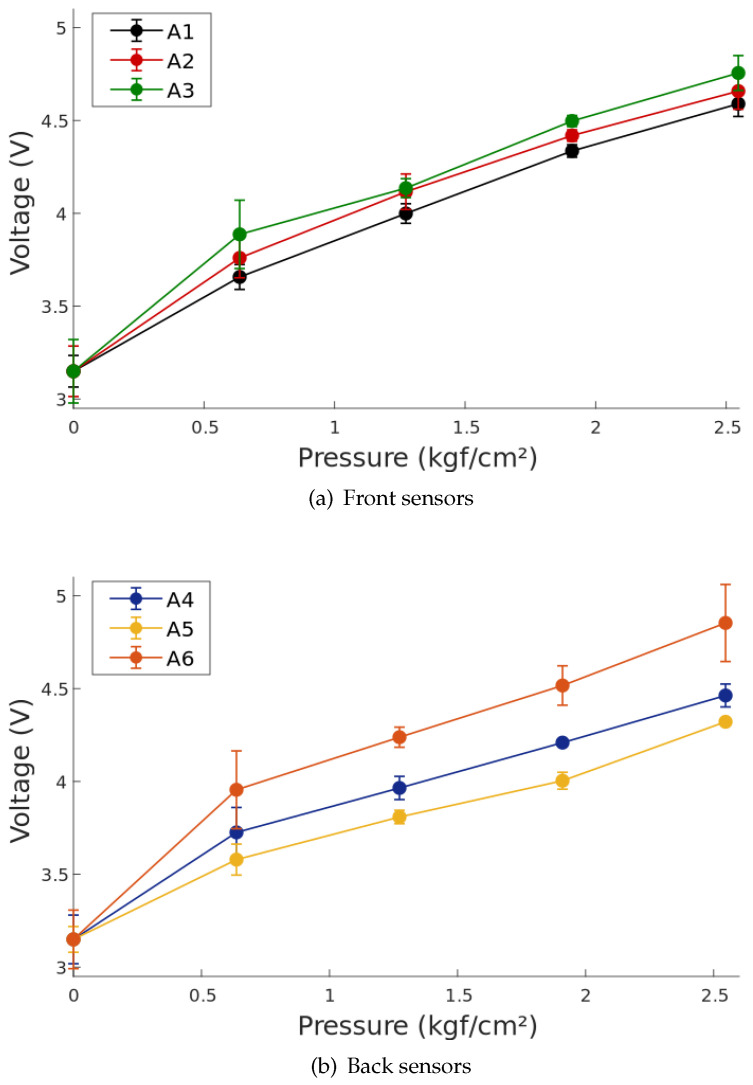
Linear Characterization of sensors showing measurement divergence. (**a**) Error points on front sensors A1, A2, and A3. (**b**) Error points on back sensors A4, A5, and A6.

Following the experimental procedure, every sensor was subjected to three different weights and the output was compared with the corresponding regression line. The error for each point was calculated to analyze the sensor behavior, and an average for all the sensors was also calculated to analyze the behavior of the system as a whole. Figure 14 shows the characterization points and errors for the six sensors. The sensor’s sensitivity, offset, and coefficient of determination were obtained from the regression lines shown in Figure 8. Table 1 summarizes the previously mentioned parameters, along with the average error for each sensor and the average parameters for the complete system.

### 3.2. Comparison Results between F-Socket System and Socket Interface Pressure Device

For the comparative test between the socket interface pressure (SIFP) device and the F-Socket, once the readings of all the iterations carried out were obtained, the pressures exerted on each sensor were filtered and analyzed.

**Figure 15:**
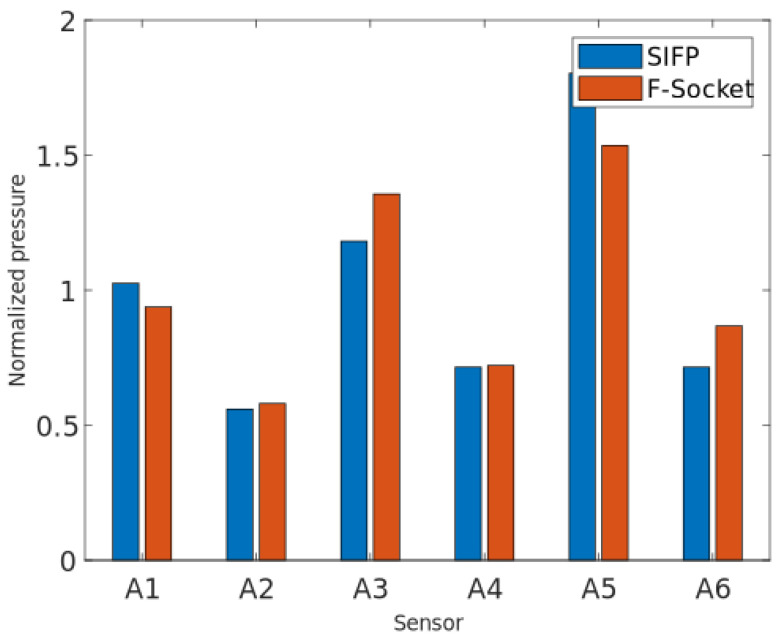
Graphical comparison of the normalized data between sensors.

The pressure magnitudes for both the SIFP sensors and the F-Socket sensors are not the same and manage to have a notable difference. These differences are expected due to the the physical characteristics of each sensor, with sensor area being the most prominent. To have a better analysis from this results, the sensor readings were normalized using the mean average of each sensor data set, as shown in Figure 15.

Once the graphs are normalized, the differences in the readings between one sensor and another can be observed, where it can be seen that, although there are some differences in the pressure readings, the SIFP device is capable of obtaining pressure readings. These differences may be due to the composition of the matrix (F-Socket) and the way it fits to the residuum.

### 3.3. SIFP System Test

In order to compare the resulting interface pressure (IP) during the gait of the participant, two tests were carried out; in the first one, the participant wore one type of patellar tendon bearing socket (PTB) identified as SD1, and, in the second test, another type of PTB socket identified as SD2. Each socket was designed and manufactured by different prothesist using conventional hand made processes. It should be noted that the participant had a transtibial amputation of the left leg.

For the tests carried out with the pressure sensors of the SIFP device, the stance gait cycle phase was the one of interest since the pressures exerted on the socket through the residuum of the participant show a peak when the prosthetic foot is in contact with the treadmill band and the socket is completely loaded with the body weight.

**Figure 16:**
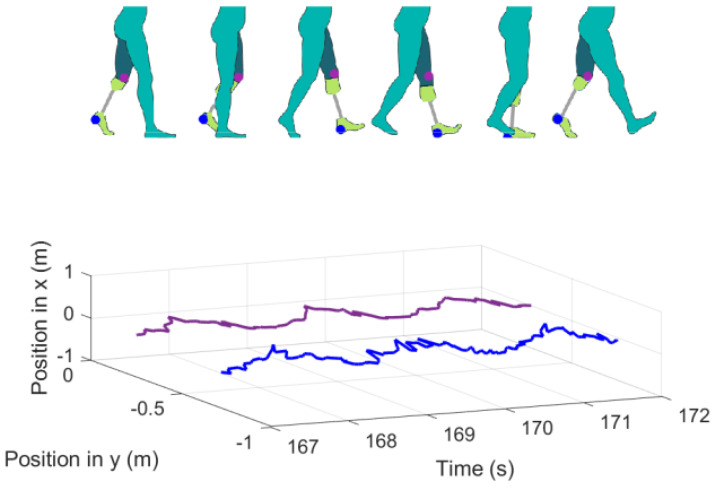
Left amputee gait cycle paired with lateral position of left knee and prosthetic foot from the second 167 to 172.

During the gait cycle of the participant, two variables from the motion capture data were chosen to display, and each variable is identified in Figure 16 with a round mark of different color. Those variables and the SIFP were recorded at the same time during the ten-minute walk.

As seen in Figure 16, the chosen variables to plot were the lateral position coordinates of the knee and prosthetic foot in space for the participant, meaning position coordinates seen in the *x* and *y* axis in time (s). Only this foot was displayed because the support phase of the prosthetic foot was synchronized in post-processing with the data collected by the SIFP device during the gait; the phase of the gait cycle is identified by a single reference lower limb, which, in this case, is the one displayed in the support phase and swing phase by the motion capture system, as seen in Figure 16.

The time lapse chosen for results analysis was from the third minute of gait because that is a moment in time in which the coordinate measurement has reached a point of stability during the motion capture. Figure 16 shows in the gait cycle image a point in blue over the prosthetic foot; this point corresponds to the ankle coordinates registered by the motion capture system in time, and the *x* and *y* change in time is noted. The purple line is the knee trajectory, also noted in the left amputee gait cycle, also shown in Figure 16. Both the knee and the prosthetic foot should show, and did show, a similar movement in the *x* axis given the hip momentum; therefore, the *x* knee trajectory is smaller due to the muted movement during the gait. In the *y* axis, the knee and foot show a parallel distance because of their anatomical position. The motion capture system saved the positions of the limbs in time to be able to synchronize them with the pressure measurements obtained from the SIFP device.

**Figure 17:**
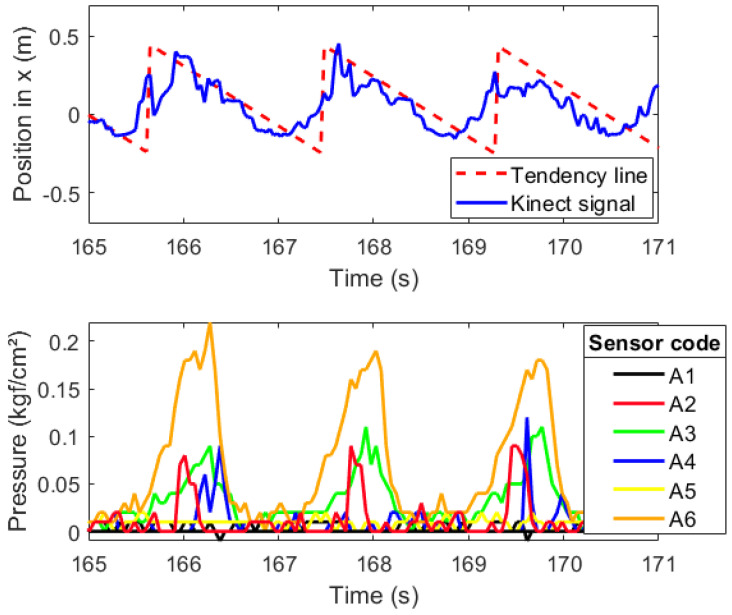
Pressure on the residuum and prosthetic leg trajectory for SD1.

For the first type of socket (SD1), the gait cycle is shown along the lateral coordinates of the knee and prosthetic foot, and Figure 17 can be read in terms of position and pressure.

In the first graph of Figure 17, the prosthetic foot trajectory is presented in blue. The trajectory shown belongs only to the *x* axis position in time. Because the participant data was obtained while walking on a treadmill, the *x* axis position does not increment linearly but oscillates in between the maximum coordinate reached by the ankle, which happens during the heel strike or initial contact to start the stance gait phase, and the minimum when the stance phase ends in the pre-swing phase when the metatarsus is about to rise from the floor. The oscillation is given by the treadmill motion. The dashed red line represents an ideal *x* axis trajectory on a treadmill setting for the ankle.

**Figure 18:**
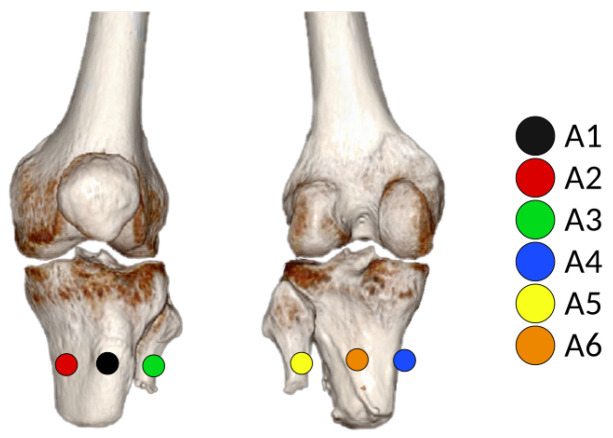
Location of sensors in the CROIs on the left residual limb.

Figure 17 shows the interface pressure distribution for the first socket (SD1), stride by stride, and the resulting peak pressures per each gait cycle noticeable in seconds 166, 168, and 169.5 noted in Table 2 are described and discussed, according to Figure 18.

During a time lapse of six seconds, the ankle trajectory and the pressure measured between the socket and residuum were plotted together in Figure 17 to check that indeed the peaks of pressure on the residuum increased as the stance phase on the gait reached its 50% is more noticeable in sensor A6 in orange shown in Table 2. This means that, in the middle of the stance phase for the prosthesis, all the body weight is carried on the prosthetic foot, which explains the peaks on interface pressure between the residuum and the socket around 0.25 kgf/cm${}^{2}$. Specifically, the A6 sensor showed the highest peak of pressure in the time lapse of 6 s (see Figure 17) that, according to CROI location, this sensor corresponds to the area below the popliteal region of the residuum, meaning that the posterior origin of the tibia and fibula press between the soft tissue and the socket favoring a possible pain and stress point due to the peak pressure (see Figure 18). Another elevated pressure point is located at the A3 sensor with a max peak of 0.11 kgf/cm${}^{2}$ where the tibial medial condyle is located, which is a bone bulge, just as the tibial lateral condyle which is around the A2 sensor, also showing a higher pressure peak.

Finally, another remarkable peak is at the back medial part of the residuum, close to the fibula condyle, another bone bulge close to the A4 sensor with a peak pressure of 0.12 kgf/cm${}^{2}$. For greater detail, in Table 2, all peak pressures for each gait cycle during the 6 s sample are broken down.

**Figure 19:**
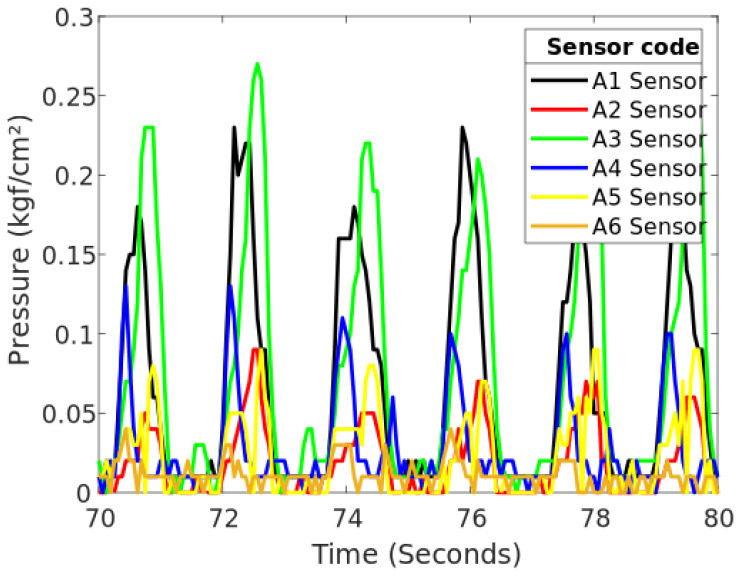
Pressure generated during the second minute of the gait for SD2.

For the second type of the socket (SD2), Figure 19 and Table 3 show the pressures that were sensed during the second minute of the gait; as it can be seen, there are sensors in which their pressure cannot be compared with the others. This is because the positions of the sensors are not the same. Another thing that could be happening is that the socket may be generating more pressure at certain points once in place. This can be visualized as a pressure spike which is generated once the participant fully reloads the foot on the treadmill.

To analyze the points that were detected with the highest pressure in more detail, only the sensors that showed this aspect during the same period of time were graphed.

**Figure 20:**
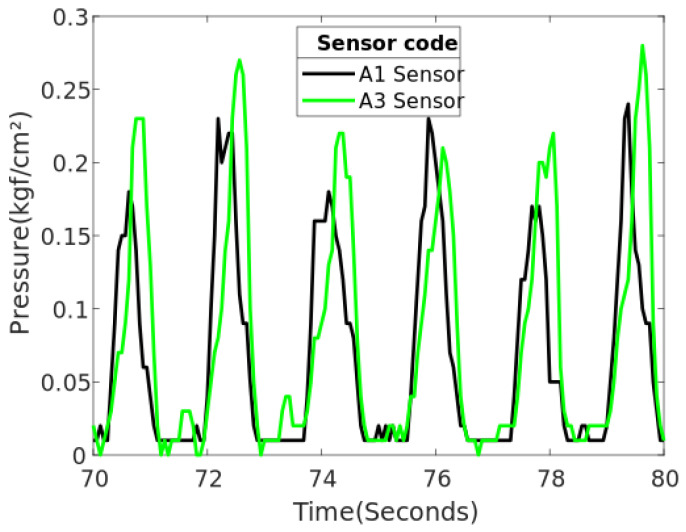
Higher pressures generated during second minute of the gait for SD2.

Once the readings of sensors A1 and A3 are plotted, it can be seen that the behavior of both sensors are very similar in terms of reading with respect to time; however, it is observed that there is a slight difference in the pressure reading. See Figure 20.

Analyzing Figure 18, on the location of the sensors in the CROI, it can be seen that these sensors that generate higher pressure are located in the front part of the residuum, on the tibial crest (A1 sensor) and in the right lateral epicondyle (A3 sensor), respectively.

## 4. Discussion

System characterization resulted in uneven parameters for each individual sensor, having individual regressions allowed for error reduction as not all sensors showed the exactly same behavior. This could be due to wear of the sensors and difference in antiquity. The system presented an almost linear behavior and an average error below 10%, permitting a correct analysis of pressure magnitude in the CROIs.

Uneven distribution of pressure between the residual limb and the prosthetic socket has a significant effect on the sensation of the amputee regarding the comfort of his prosthesis. In this research work, a SIFP system was developed to measure load distribution within the socket. This system will allow having a quantitative measure of the quality fit of the socket for lower limb prosthetic users, beyond an exit survey that qualitative measures amputee’s opinion. The sensor localized in CROI A1 for the SD2 sensed high pressure in the front part of the residuum, on the tibial crest area, which indicates a possible pain point, and this result is similar to the results presented by Zhang et al. [17]. Similar systems, such as the Tekscan or the F-Socket system, are currently commercially available. However, it was determined that it is difficult to use these systems to measure the loads within the socket due to the irregular shape of the lower limb volume.

The socket interface pressure system developed in this research work makes possible the measurement of pressure at the CROI location, as well as the analysis of the load distribution between sockets. The developed system was validated against the commercially available F-Socket system. The distribution of loads was found to be similarly identified by both systems, verifying that the developed system can identify load differences in the CROIs. In addition, obtained pressure data was compared to the gait cycle recorded with the motion capture system, corroborating the loads inside the socket with the gait phases observed. This system could be used in conjunction with other systems to analyze the performance and comfort of different types of sockets, as well as quantitatively determine the improvement of one over another.

The intended purpose of this system was to use it to identify the part of the gait cycle the participant was in, during a walk on a treadmill at 1.4 m/s speed at a self-selected cadence. The prosthetic foot was selected as the reference to analyze the gait cycle part by watching the foot trajectory in a one-dimensional setting in the *X-axis* and a two-dimensional setting on the *X-axis* and *Y-axis* since the gait cycle was repeated during the ten-minute motion capture over the treadmill.

In other studies regarding the interface pressure, the F-Socket, as mentioned above, was used as the pressure measuring system. The configuration of the sensors was set on top of the complete anterior, posterior, lateral, and medial areas of the remaining limb [19], while the use of CROI as the sensors placement was chosen in this study. The Kistler force plate, used in another study for interface pressure analysis as a motion capture system, showed great practicality to identify the part of the gait cycle on a walkway, making it possible to look for a pressure pattern on certain parts of the gait cycle, showing the greatest peaks on the stance phase for the prosthetic foot [19]. A similar version of this pressure pattern is shown in Figure 17, which also shows peak pressures on the stance phase with similar shaped peaks on the anterior, posterior, lateral, and medial located CROI.

## 5. Conclusions

The socket interface pressure (SIFP) device herein proposed is able to measure, both wirelessly and in real time, a pressure profile for the interface between the socket and the residuum of a lower limb amputee during gait testing. The comparative study between the SIFP device and the F-Socket shows that the performance of the two devices in measuring interface pressures is comparable due that both of them show a similar pressure change pattern when loads were applied to an artificial residuum, thereby simulating the walking protocol. While the F-Socket system has many more active sensors, it unfortunately does not conform well to the irregular shape of the residuum as does the SIFP device.

The SIFP system serves to evaluate the functional performance of prosthetic sockets using the residuum/socket pressure distribution as shown during the donning of a unilateral transtibial amputee during a 10-min gait cycle test. The experiment also showed that pressures have significant changes when the participant uses a socket manufactured with a different technique. This finding could further help to expand the study in a larger sample of participants and evaluate differently-manufactured sockets for the transtibial amputees and possibly identify anomalies in posture and gait. Finally, to complete the comfort analysis for a socket, in addition to studying the interface pressure, gait cycle and bio-mechanical variables, a satisfaction survey could provide an overview on the comfort of the evaluated socket, as well as to help perceive areas for improvement from the user’s perspective [20].

This SIFP system may be used by prosthetists, physical therapists, amputation care centers, and researchers, as well as government and private regulators requiring comparison and evaluation of prosthetic components, components under development, and testing.

## Figures and Tables

**Table 1:** Pressure sensor static characterization results.

Sensor ID	Sensibility	Offset	R²	Av Error (%)
**A1**	0.5591	3.2344	0.9842	6.13%
**A2**	0.5775	3.2552	0.9647	9.34%
**A3**	0.6006	3.3203	0.951	10.59%
**A4**	0.4886	3.2803	0.9576	7.87%
**A5**	0.4349	3.2188	0.9806	4.79%
**A6**	0.6888	3.3071	0.9206	14.70%
**Average**	**0.5475**	**3.2813**	**0.9654**	**8.00**%

**Table 2:** Peak pressures recorded by the sensors in each cycle of the gait for SD1.

	Pressure Peaks per Gait Cycle					
**Sensors** **Gait Cycle**	**A1**	**A2**	**A3**	**A4**	**A5**	**A6**
1	0.08 kgf/cm${}^{2}$ (7.84 kPa)	0.02 kgf/cm${}^{2}$ (1.96 kPa)	0.09 kgf/cm${}^{2}$ (8.83 kPa)	0.08 kgf/cm${}^{2}$ (7.84 kPa)	0.02 kgf/cm${}^{2}$ (1.96 kPa)	0.22 kgf/cm${}^{2}$ (21.57 kPa)
2	0.09 kgf/cm${}^{2}$ (8.83 kPa)	0.02 kgf/cm${}^{2}$ (1.96 kPa)	0.11 kgf/cm${}^{2}$ (10.79 kPa)	0.02 kgf/cm${}^{2}$ (1.96 kPa)	0.02 kgf/cm${}^{2}$ (1.96 kPa)	0.19 kgf/cm${}^{2}$ (18.63 kPa)
3	0.09 kgf/cm${}^{2}$ (8.83 kPa)	0.02 kgf/cm${}^{2}$ (1.96 kPa)	0.11 kgf/cm${}^{2}$ (10.79 kPa)	0.12 kgf/cm${}^{2}$ (11.77 kPa)	0.02 kgf/cm${}^{2}$ (1.96 kPa)	0.18 kgf/cm${}^{2}$ (17.65 kPa)

**Table 3:** Peak pressures recorded by the sensors in each cycle of the gait for SD2.

	Pressure Peaks per Gait Cycle					
**Sensors** **Gait Cycle**	**A1**	**A2**	**A3**	**A4**	**A5**	**A6**
1	0.18 kgf/cm${}^{2}$ (17.65 kPa)	0.23 kgf/cm${}^{2}$ (22.55 kPa)	0.18 kgf/cm${}^{2}$ (17.65 kPa)	0.23 kgf/cm${}^{2}$ (22.55 kPa)	0.17 kgf/cm${}^{2}$ (16.67 kPa)	0.24 kgf/cm${}^{2}$ (23.53 kPa)
2	0.05 kgf/cm${}^{2}$ (4.90 kPa)	0.09 kgf/cm${}^{2}$ (8.83 kPa)	0.05 kgf/cm${}^{2}$ (4.90 kPa)	0.07 kgf/cm${}^{2}$ (6.86 kPa)	0.07 kgf/cm${}^{2}$ (6.86 kPa)	0.06 kgf/cm${}^{2}$ (5.88 kPa)
3	0.23 kgf/cm${}^{2}$ (22.55 kPa)	0.27 kgf/cm${}^{2}$ (26.47 kPa)	0.22 kgf/cm${}^{2}$ (21.57 kPa)	0.21 kgf/cm${}^{2}$ (20.59 kPa)	0.20 kgf/cm${}^{2}$ (19.61 kPa)	0.28 kgf/cm${}^{2}$ (27.45 kPa)
4	0.13 kgf/cm${}^{2}$ (12.74 kPa)	0.13 kgf/cm${}^{2}$ (12.74 kPa)	0.11 kgf/cm${}^{2}$ (10.79 kPa)	0.10 kgf/cm${}^{2}$ (9.80 kPa)	0.10 kgf/cm${}^{2}$ (9.80 kPa)	0.10 kgf/cm${}^{2}$ (9.80 kPa)
5	0.08 kgf/cm${}^{2}$ (7.84 kPa)	0.09 kgf/cm${}^{2}$ (8.83 kPa)	0.08 kgf/cm${}^{2}$ (7.84 kPa)	0.07 kgf/cm${}^{2}$ (6.86 kPa)	0.09 kgf/cm${}^{2}$ (8.83 kPa)	0.09 kgf/cm${}^{2}$ (8.83 kPa)
6	0.04 kgf/cm${}^{2}$ (3.92 kPa)	0.03 kgf/cm${}^{2}$ (2.94 kPa)	0.03 kgf/cm${}^{2}$ (2.94 kPa)	0.04 kgf/cm${}^{2}$ (3.92 kPa)	0.02 kgf/cm${}^{2}$ (1.96 kPa)	0.02 kgf/cm${}^{2}$ (1.96 kPa)
